# Insulin-Sensitizer Effects of Fenugreek Seeds in Parallel with Changes in Plasma MCH Levels in Healthy Volunteers

**DOI:** 10.3390/ijms19030771

**Published:** 2018-03-08

**Authors:** Rita Kiss, Katalin Szabó, Rudolf Gesztelyi, Sándor Somodi, Péter Kovács, Zoltán Szabó, József Németh, Dániel Priksz, Andrea Kurucz, Béla Juhász, Zoltán Szilvássy

**Affiliations:** 1 Department of Pharmacology and Pharmacotherapy, Faculty of Medicine, University of Debrecen, H-4032 Debrecen, Hungary; kiss.rita@med.unideb.hu (R.K.); szabo.katalin@pharm.unideb.hu (K.S.); gesztelyi.rudolf@pharm.unideb.hu (R.G.); nemeth.jozsef@med.unideb.hu (J.N.); priksz.daniel@pharm.unideb.hu (D.P.); kurucz.andrea@pharm.unideb.hu (A.K.); szilvaszilva99@gmail.com (Z.S.); 2 Department of Internal Medicine, Faculty of Medicine, University of Debrecen, H-4032 Debrecen, Hungary; somodi@belklinika.com (S.S.); kovacsp@belklinika.com (P.K.); 3 Department of Emergency Medicine, Faculty of Medicine, University of Debrecen, H-4032 Debrecen, Hungary; szaboz.med@gmail.com

**Keywords:** hyperinsulinemic euglycemic glucose clamp (HEGC), fenugreek, melanin-concentrating hormone (MCH), RIA, type 2 diabetes, clinical pilot study

## Abstract

In developed, developing and low-income countries alike, type 2 diabetes mellitus (T2DM) is one of the most common chronic diseases, the severity of which is substantially a consequence of multiple organ complications that occur due to long-term progression of the disease before diagnosis and treatment. Despite enormous investment into the characterization of the disease, its long-term management remains problematic, with those afflicted enduring significant degradation in quality-of-life. Current research efforts into the etiology and pathogenesis of T2DM, are focused on defining aberrations in cellular physiology that result in development of insulin resistance and strategies for increasing insulin sensitivity, along with downstream effects on T2DM pathogenesis. Ongoing use of plant-derived naturally occurring materials to delay the onset of the disease or alleviate symptoms is viewed by clinicians as particularly desirable due to well-established efficacy and minimal toxicity of such preparations, along with generally lower per-patient costs, in comparison to many modern pharmaceuticals. A particularly attractive candidate in this respect, is fenugreek, a plant that has been used as a flavouring in human diet through recorded history. The present study assessed the insulin-sensitizing effect of fenugreek seeds in a cohort of human volunteers, and tested a hypothesis that melanin-concentrating hormone (MCH) acts as a critical determinant of this effect. A test of the hypothesis was undertaken using a hyperinsulinemic euglycemic glucose clamp approach to assess insulin sensitivity in response to oral administration of a fenugreek seed preparation to healthy subjects. Outcomes of these evaluations demonstrated significant improvement in glucose tolerance, especially in patients with impaired glucose responses. Outcome data further suggested that fenugreek seed intake-mediated improvement in insulin sensitivity correlated with reduction in MCH levels.

## 1. Introduction

### 1.1. Major Pathologic Features of T2DM

Type 2 diabetes mellitus, T2DM or NIDDM (non-insulin-dependent diabetes mellitus) is a metabolic disorder characterized by hyperglycemia, insulin resistance and relative insulin deficiency. Its primary pathomechanism is persistent hyperglycemia, due to inability of a subject’s cells to induce adequate metabolic responses to insulin produced by pancreatic β islet cells. A major feature of the disease is the inability of an individual’s insulin to maintain normoglycemia. Major consequences occurring due to reduced insulin sensitivity by tissues for which glycemic balance is particularly important, such as adipose tissue muscles, liver, may be very severe [1,2,3,4,5,6].

### 1.2. Mechanisms Contributing to T2DM Onset And Progression

T2DM is often associated with obesity and pathologically altered lipid metabolism [7]. The pathogenesis of the disorder also involves aberrant alteration in levels and function of many centrally or peripherally acting hormones and peptides, including: insulin, leptin, ghrelin, glucagon-like peptide-1 (GLP-1), melanin-concentrating hormone (MCH) and many others. T2DM is the most common form of diabetes with a proportion of 90–95% compared to T1DM [8]. Its incidence is gradually growing, and professionals and organizations, including the World Health Organization (WHO) and the American Diabetes Association predict that by 2030 we will face a kind of “diabetes epidemic” [2,4,9,10,11].

### 1.3. Countermeasures to T2DM Progression

This diabetic condition usually can be controlled by lifestyle changes, including general diet, use of dietary supplements and/or oral antidiabetic agents [12,13,14,15]. The objective of these strategies, which is to normalize blood glucose, is increasingly aided by identification of agents based on generally regarded as safe (GRAS) plant materials, some of which, such as fenugreek (*Trigonella foenum graecum*/TFG), are generally widely represented in the human diet. Fenugreek is one of the oldest medicinal plants, endemic to India and North Africa [16,17]. Currently it is widely cultivated worldwide, especially in the Mediterranean region. Both seeds and leaves of fenugreek are widely used as a culinary spice (to enhance the taste of many meat or vegetable dishes) [18,19]. The seeds are used as a therapeutic supplement for numerous indications: as an appetite stimulant, to treat digestive disorders, to increase milk production in lactating women and treatment of hyperlipidemia and diabetes [20,21]. The major bioactive components of fenugreek seeds include: alkaloids, amino acids, flavonoids, fibers, coumarin, lipids, vitamins, minerals, notable saponins and steroidal sapogenins, of which those of most intensive interest, are yamogenin and diosgenin [16,22,23]. Fenugreek seeds possess well known therapeutic effects, the most important of which include their antidiabetic activity, related regulation of food intake and mood disorders. They additionally exhibit antioxidant, hypolipidemic, cardioprotective and gastroprotective effects, immunoregulatory, antimicrobial, anti-inflammatory activities and analgesic properties. Moreover, recent studies of the seeds demonstrate that their components are agonists on the MCHR1 isoform of MCHRs (melanin-concentrating hormone receptor) [18,24,25,26,27].

### 1.4. MCH Properties

MCH is a cyclic peptide that was first isolated and purified from salmon pituitary gland and structurally characterized by Kawauchi et al., in 1983 [28]. Originally, its primary biological function was considered to be skin paling in fish [29,30]. Further investigation revealed mammalian MCH expression, with potential for a diverse range of functions. In mammals, MCH is a nonadecapeptide produced in the hypothalamus and secreted predominantly in neurons of the lateral hypothalamic area (LHA) and zona incerta (ZI) in the subthalamus. Moreover, its presence has been demonstrated in peripheral tissues, including pancreatic β-cells, colonic epithelial cells and adipocytes [31,32,33,34,35,36].

### 1.5. MCHR Distribution and Major Activities

The effects of MCH are mediated by two receptors belonging to the G-protein-coupled receptor family: MCH-R1 and MCH-R2 [30]. Expression of MCH-R1 is the highest in the ventromedial and dorsomedial nuclei of the hypothalamus, and both appear to be regulators of food intake [37]. Additional roles for these hormones have been shown in diabetes, obesity, and gastrointestinal and mood disorders. In most sources, this isoform exhibits approximately 38% homogeneity with MCH-R1 [29,30,38]. The biological function of MCH-R2 remains undefined at the time of this writing, but its wide expression in the brain and expression patterns in peripheral tissues, suggest diverse regulatory functions [39,40]. Investigation of this molecule has additionally revealed that MCH-R2 expression is different than that of the MCH-R1 isoform in certain areas of the brain, including olfactory tissue, nucleus accumbens, amygdala along with hippocampal, hypothalamic cells. MCH-R1 expression is typically limited to the cortical area of the brain and it is not present in the periphery [41].

### 1.6. Differential Effects of MCH and Relevance to Diabetes Research

Accordingly, stimulation of each of these two isoforms is expected to have differential effects on neurologic function and downstream effects (that may possibly be in the opposite direction) [41,42]. MCH thus appears to be an important mediator of metabolic processes, food intake, energy expenditure, obesity, mood control, stress and sleep-wake cycles. Intriguingly, in the context of the investigation described by this report, recent studies have demonstrated a role for MCH in the regulation of pancreatic beta-cell function [43,44,45,46,47], findings which led the authors of this report to speculate that bioactive components of fenugreek seeds may exert medically useful effects on these processes.

### 1.7. Objectives of Present Investigation

The study described in the present report was undertaken to evaluate a hypothesis that an orally administered preparation of fenugreek seeds reduces hyperglycemia through insulin sensitization. The investigation further assessed the safety and tolerance of repetitive short interval administration of fenugreek seed preparation for a given subject during a ten-day period. A corollary hypothesis tested in the present study is that melanin-concentrating hormone (MCH) is a critical component of T2DM-associated changes in insulin sensitization.

## 2. Results

### 2.1. Fasting Serum Glucose Levels in Placebo- and Fenugreek-Treated Subjects

**Figure 1:**
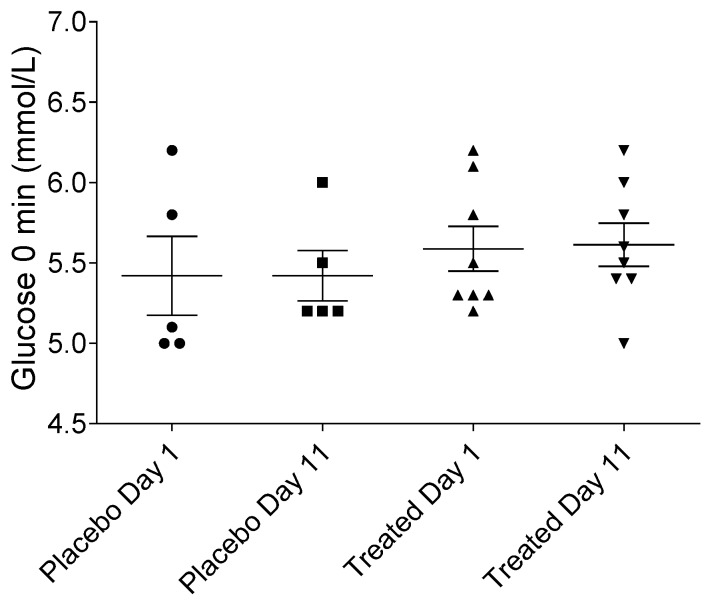
Fasting serum glucose levels in the placebo- and fenugreek-treated subjects. No significant differences were found among groups.

To adjust the rate of glucose infusion, it was necessary to determine the fasting blood glucose levels. As expected, there were no statistically significant differences in the fasting serum glucose levels among the fenugreek- or placebo-treated groups (Figure 1).

### 2.2. Fasting Plasma Insulin Levels of the Placebo- and Fenugreek-Treated Subjects

**Figure 2:**
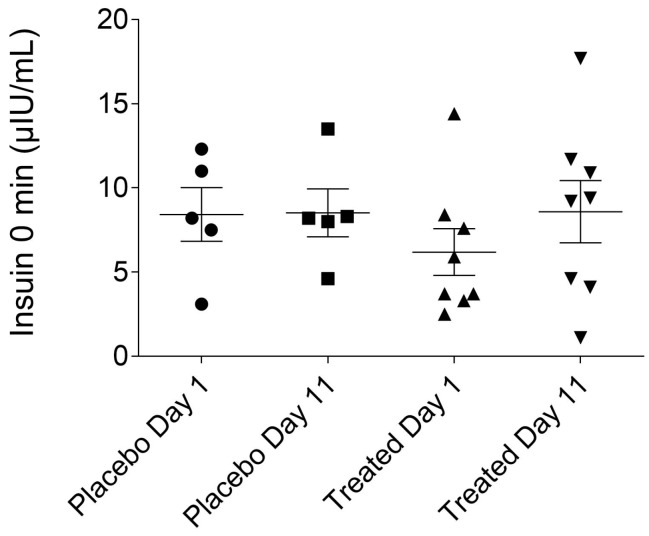
Fasting plasma insulin levels of the placebo- and fenugreek-treated subjects.

Determining the fasting insulin level is necessary to calculate derived parameters and also for the safe execution of HEGC procedure. The baseline insulin levels were in the normal ranges for all of the volunteers, and no significant differences were found between the fasting plasma insulin levels of the fenugreek- and placebo-treated groups (Figure 2).

### 2.3. Effects of TFG on Glucose Infusion Rate (GIR)

**Figure 3:**
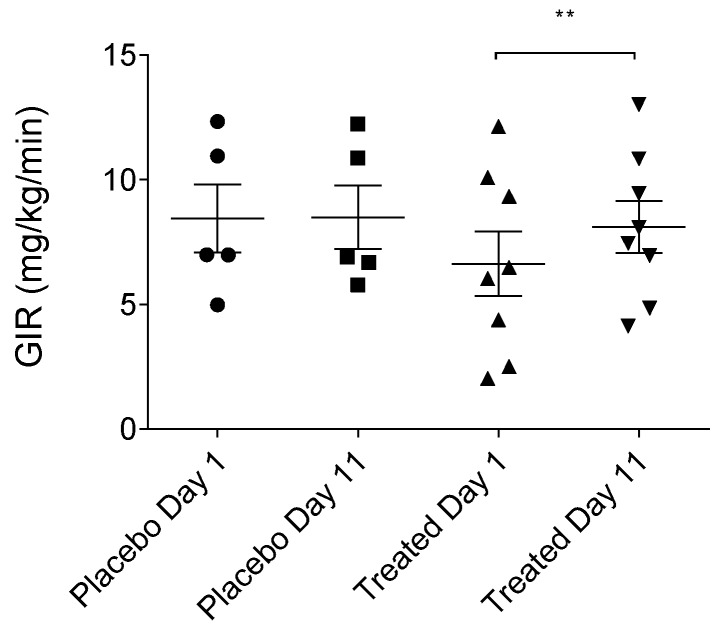
GIR in the placebo- and fenugreek-treated subjects. ** refers to statistically significant difference (*p* < 0.01) between Day 1 and Day 11 in the treated group.

During the hyperinsulinemic euglycemic glucose clamp (HEGC) examination, the steady-state glucose infusion rate significantly increased among the fenugreek-treated volunteers, referring to the insulin-sensitizing effect of the TFG (Table 1 and Figure 3). It is notable that GIR values of the TFG-treated group were significantly lower at the baseline point (before the initiation of the treatment). Although, patients were randomly divided into groups, two subjects with diminished GIR were sorted to the TFG group, as it turned out during the HEGC procedure. Interestingly, TFG-treatment exerted the most powerful effect in these two cases (increasing GIR up to 100%).

### 2.4. Serum Lipid Levels of the Placebo- and Fenugreek-Treated Subjects

**Figure 4:**
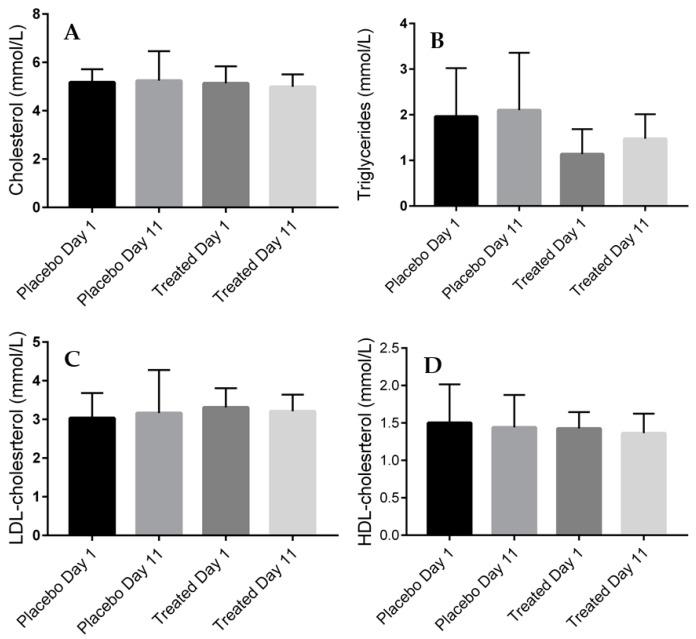
Serum lipid levels of the placebo- and fenugreek-treated subjects. (**A**) serum total-cholesterol levels (mmol/L); (**B**) serum triglyceride levels (mmol/L); (**C**) high-density lipoprotein (HDL)-cholesterol levels (mmol/L); (**D**) low-density lipoprotein (LDL)-cholesterol levels (mmol/L).

Figure 4 represents our results showing no statistically significant differences in the serum lipid parameters (total cholesterol, triglycerides, low-density lipoprotein (LDL) and high-density lipoprotein (HDL)-cholesterol) of the fenugreek- and placebo- treated groups.

### 2.5. Calculated Parameters of Insulin Sensitivity Assessment (MCRI, ISI, QUICKI, HOMA-IR and HOMA-%β) in the Placebo- and Fenugreek-Treated Subjects

**Figure 5:**
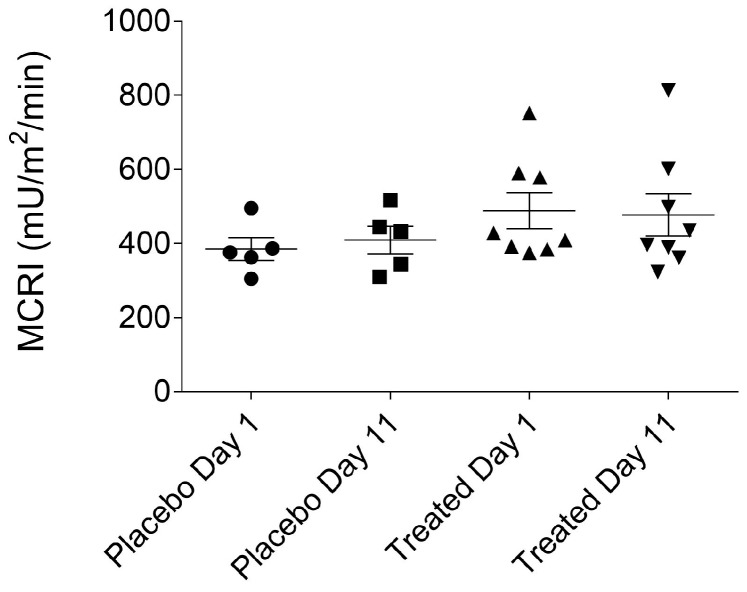
MCRI (metabolic clearance rate of insulin) of the placebo- and fenugreek-treated subjects.

**Figure 6:**
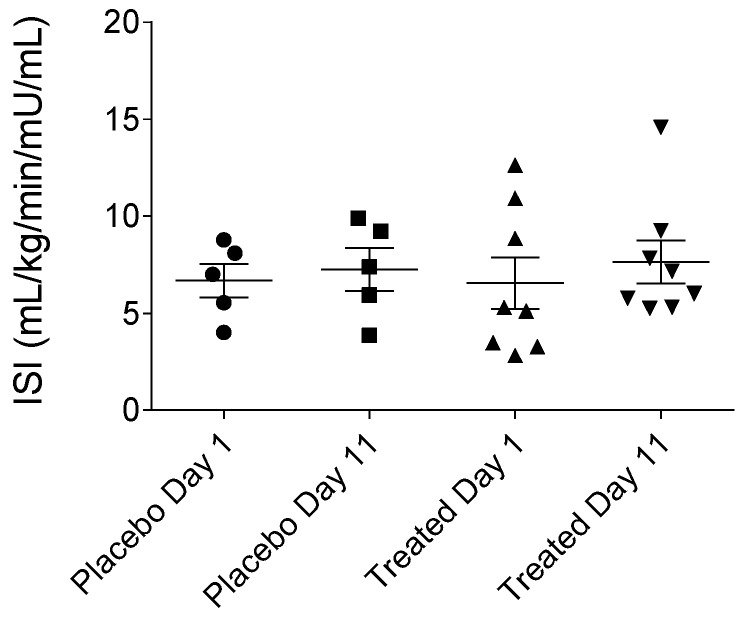
Insulin sensitivity index of the placebo- and fenugreek-treated subjects.

**Figure 7:**
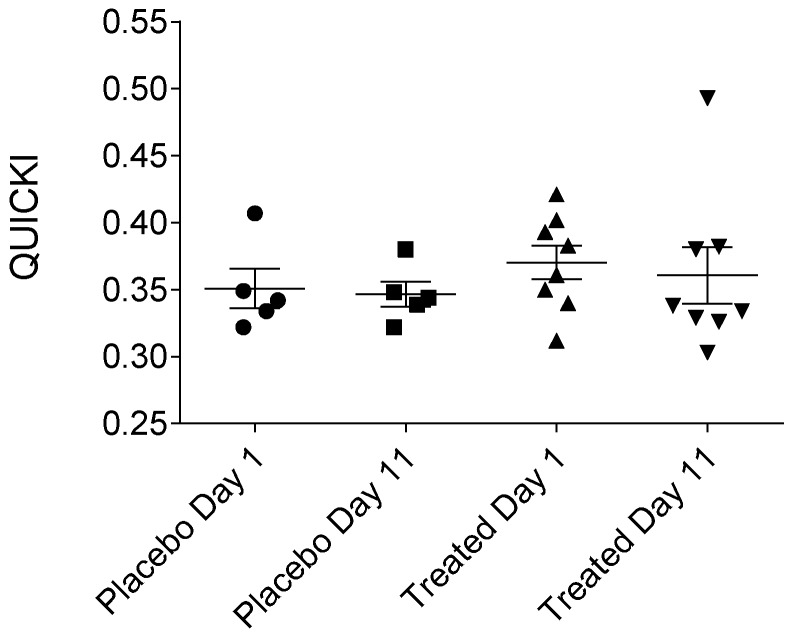
QUICKI (quantitative insulin sensitivity check index) of the placebo- and fenugreek-treated subjects.

**Figure 8:**
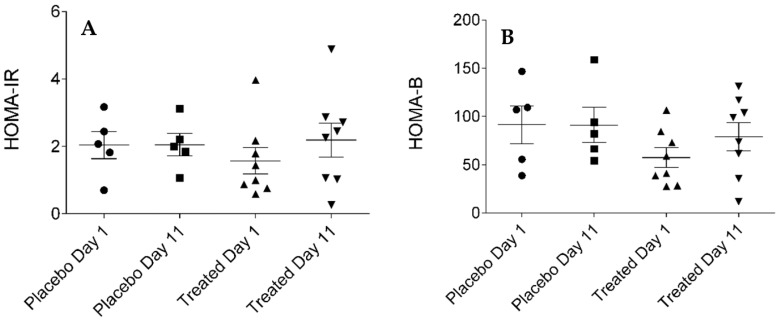
HOMA-IR (homeostatic model assessment-insulin resistance, (**A**) and HOMA-B (homeostatic model assessment-beta cell function, (**B**) of the placebo- and fenugreek-treated subjects.

MCRI (metabolic clearance rate of insulin), ISI (insulin sensitivity index), QUICKI (quantitative insulin sensitivity check index), HOMA-IR (homeostatic model assessment-insulin resistance) or HOMA-B (homeostatic model assessment-beta-cell function) indices were calculated to precisely assess insulin sensitivity. Our results presented in Figure 5, Figure 6, Figure 7 and Figure 8, show that no statistically significant difference could be observed in the values of the abovementioned indices between the fenugreek- and placebo-treated groups. Steady-state insulin levels used for calculations are presented in Supplementary Material section (Supplementary Table S5) of this paper.

### 2.6. Effect of TFG on Plasma MCH Levels

**Figure 9:**
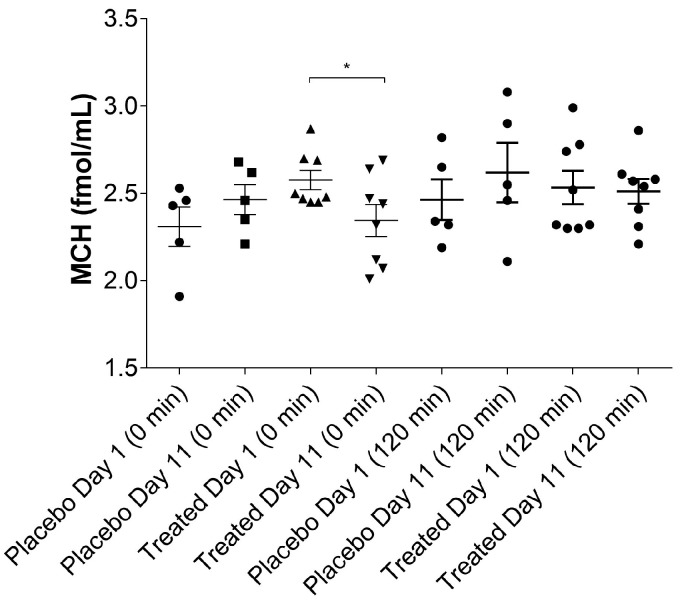
MCH (melanin-concentrating hormone) levels of the placebo- and fenugreek-treated subjects. * represents significant difference (*p* < 0.05) between Day 1 and Day 11 in the treated group.

Plasma MCH concentrations were measured by a highly specific and sensitive radioimmunoassay (RIA) method developed recently by our team, as is described in Section 4.8. Our assumption is partially confirmed by the fact that after ten days of Fenugreek treatment, plasma MCH concentration was significantly reduced in the TFG-treated group, when measured prior to HEGC (0 min sample). On the contrary, no statistically significant differences were found in the MCH plasma concentrations after 120 min of the HEGC procedure, as shown in Figure 9.

### 2.7. Correlation between MCH and Serum Lipid Levels

Analyzing the relationship between plasma MCH concentration and serum lipid levels of fenugreek-treated volunteers, we observed that elevation of plasma MCH level may be associated with a decreasing concentration of total cholesterol, including LDL- and HDL-cholesterol, and although the differences do not reach the level of statistical significance, a clear tendency can be seen (Supplementary Tables S1 and S2). In parallel, we showed a negative correlation between the MCH and triglyceride levels. Data is presented in Supplementary Material, in Supplementary Tables S1 and S2.

### 2.8. Correlation between MCH and Serum Glucose Levels

Analyzing the correlation between the peripheral expression of MCH and serum glucose concentrations we did not observe any statistically significant association between the MCH and glucose levels in placebo- and fenugreek-treated volunteers (data in Supplementary Table S3).

### 2.9. Correlation between MCH and Plasma Insulin Levels

Our results indicate that no statistically significant correlation could be detected between the MCH and insulin plasma concentration (data presented in Supplementary Table S4).

## 3. Discussion

Outcomes of the experiments conducted in the present investigation demonstrate that fenugreek seed does indeed exhibit insulin-sensitizing effects in tissues. Evidence for this conclusion is based on observations that glucose infusion rate (GIR) values significantly increased in participating subjects, following ten-day treatment periods with fenugreek seed preparations (*p* = 0.001327). The largest increases in GIR, corresponding to an insulin sensitizing effect of 97%, occurred in two patients with the lowest GIR values in the test group, measured before initiation of treatment. GIR increases were also observed in the other six volunteers treated with fenugreek capsules, however, to a much lesser extent than in the two subjects with the lowest measured GIRs. In contrast, placebo-treated subjects, failed to exhibit significant change in GIR values. According to the abovementioned findings, we may conclude that the TFG-treatment is more effective when the patient is insulin-resistant, and that it does not have influence on glucose homeostasis under normal, physiological conditions. Additionally, no significant placebo-associated changes were observed in MCRI, QUICKI, ISI, HOMA-IR, and HOMA-B, serum glucose, plasma insulin and serum lipids. Interpretation of these data is, nevertheless, subject to the caveat that the present study is a preliminary analysis of fenugreek seed effects and not intended as a definitive analysis of T2DM pathomechanism. Here, the sample ‘n’ for test subjects is low, and it is anticipated that more comprehensive future studies will involve participation of larger groups of volunteers. Nevertheless, results of this small pilot study are encouraging, since safety and efficacy of fenugreek seeds have been proven in human subjects, with parallel changes in the plasma level of MCH, a mediator that may play an important role in glucose-homeostasis. Future clinical and basic lab analyses of this biomaterial, planned by the authors, will extend the experiments described here into detailed characterization of signaling processes contributing to insulin sensitization that are sensitive to modulation by fenugreek and other natural products. Such investigations will be conducted in tandem with evaluations of currently available insulin-sensitizing drugs to determine the capability of natural substances, to potentiate their pharmacological value and reduce occurrence of side effects. Haines et al. have previously conducted product development research that achieved these objectives [48]. A major outcome of the present study included an observation that following ten days of fenugreek seed ingestion, serum MCH levels were significantly reduced (*p* = 0.0464), while no such reduction was observed in the placebo-treated group. These results demonstrate that fenugreek-mediated augmentation of insulin sensitization and improved glucose metabolism correlate with and may be substantially dependent on decrease of MCH levels. The MCH is involved in several physiological functions in vertebrates. Evidence suggests its crucial role in energy homeostasis, food intake, obesity and T2DM. The increased levels of MCH is correlated with hyperphagia, body weight gain, increased white adipose tissue and liver mass, reduce brown adipose tissue function, moreover, elevated MCH may produce hyperglycemia, hyperinsulinemia, and hyperleptinemia [49,50,51,52,53,54]. On the contrary, MCH-knockout mice are lean and resistant to obesity, furthermore, resistant to age-related glucose intolerance [55]. In the periphery, MCH was detected in several tissues, e.g., in the beta cells of the pancreatic islets, adipose tissues and in the duodenum. MCH induces islet hyperplasia, which suggest that it may regulate pancreatic islet secretory function and beta cell dynamics [35,36,56,57]. Moreover, Pereira-da-Silva et al. suggested that MCH may act as the central modulator of insulin activity, as it could modify glucose metabolism [45]. MCH-containing neurons and MCH receptors have a critical role in energy homeostasis. These (second order and downstream) neurons regulated by POMC (proopio-melanocortin) and NPY/AGRP (neuropeptide Y/agouti-related peptide) (first-order) neurons and modulate food intake. First order neurons can process the signals arriving from the periphery like ghrelin, insulin and leptin. NPY/AGRP will stimulate and POMC will inhibit MCH-containing neurons. Ghrelin act as a positive modulator on NPY/AGRP; thus, if the MCH levels will increase, food intake will be enhanced. Effects of insulin and leptin are antagonistic, whereby POMC neurons will be stimulated by these hormones, thus, MCH-containing neurons will be inhibited and food intake will decrease. MCH-containing neurons are negatively affected by autoregulation as well [58]. Furthermore, MCH-containing neurons not only secrete MCH, but also produce GABA (gamma-amino butyric acid), CART (cocaine- and amphetamine-regulated transcript) and nesfatin. These neurons can be directly activated by orexinergic neurons, and indirectly by glutamate release from excitatory fibers. In contrast, MCH inhibits orexinergic neurons and the neighboring GABAergic fibers. Furthermore, MCH-containing neurons may be inhibited by MCH, GABA, NE (norepinephrine, mediated by alpha-2 receptors), serotonin, acetylcholine (muscarinic), neuropeptide Y and histamine [59]. Interestingly, Hausen et al. found that insulin can act directly on MCH-containing neurons, which resulted in impairment of locomotor activity and insulin sensitivity [60]. This is a reasonable interpretation of the data, since elevated levels of the hormone are associated with increase in the food intake and, thus, body weight, and with an increased rate of preadipocyte differentiation [61].

To investigate the correlation between the MCH and glucose, as well as lipid metabolism, we measured the blood levels of the correspondent parameters. Although literature data presents an important decrease in the aforementioned blood parameters after fenugreek treatment, it is important to highlight the major differences between these study designs and our experimental concept. Accordingly, numerous experimental and clinical reports are available, but all enrolled patients with impaired glucose tolerance, prediabetes or manifest T2DM, while in our study, only healthy volunteers were examined [8,62,63]. Another important difference was that our volunteers were treated with a lower daily dose of TFG, 3 g/day instead of 10 g/day used by others [8,62,63]. Finally, we treated the volunteers only for ten days, in particular interest to short-term effects of TFG-treatment [8,62,63]. Probably due to these differences, we failed to find significant changes in fasting glucose, insulin or serum lipid concentrations. However, to our knowledge, this is the first study to demonstrate that fenugreek seeds may contribute to the regulation of glucose metabolism by restoring the insulin sensitivity of peripheral tissues, shown by the most sensitive method (HEGC) on human volunteers. Moreover, we observed that the fasting insulin level is higher in the TFG-treated group at the end of the study, probably due to the insulinotropic effect of the fenugreek seeds [64,65,66].

Another novel finding is that MCH-like immonoreactivity, measured by a highly specific method developed by our team, increased significantly in the treated group after ten days of treatment, when measured from blood samples at the 0 time point, before HEGC was carried out. This difference was blurred away when measuring MCH levels after the HEGC procedure, nevertheless, a possible explanation could be that serum levels of MCH—which is an orexigenic hormone responsible for energy homeostasis—alter when glucose is administered during the HEGC procedure. We consider baseline levels more reliable, and the decrease observed in the treated group is more convincing, especially if we count the fact that fasting increases MCH serum levels, as was shown by Gavrila et al. [31]. According to the preliminary results shown here, we conclude that it would be valuable to investigate effects of TFG on MCH levels in a larger number of patients who manifest T2DM. MCH-related signal transduction pathways could become new molecular targets of drug discovery and development in the future.

## 4. Experimental Section

### 4.1. Study Design

The study was a double-blind, multiple-dose, randomized, placebo controlled, single treatment period pilot study, entitled: “A pilot clinical study in healthy volunteers of the ten days multiple dose (three times daily 1000 mg) administration of Fenugreek capsules (the Hungarian product: Dr. Makai Görögszénamag) to evaluate the suspected insulin sensitizing effect behind the glycemic control of the treatment well-established in animal and human trials (Pilot study)”. The study was conducted at the Clinical Pharmacology Department at the University of Debrecen in the period 31 January–20 February 2017 and was sponsored by Libafood-Tech KFT Hungary (Debrecen, Hungary).

### 4.2. Ethical Considerations

The Clinical Study Protocol was authorized by the National Office of the Chief Medical Officer (Hungary) (IF-911-2/2017, dated: 11 January 2017) based on the Ethical Approval of the Medical Research Council—Scientific Research Ethics Committee (Hungary) of 9 January 2017 (ETT TUKEB: 1930-1/2017/EKU). The protocol was performed in compliance with International Council on Harmonization (ICH) Guidelines for Good Clinical Practices, and with the principles of the Helsinki Declaration.

### 4.3. Subject Recruitment

The volunteers enrolled in the study were recruited from students, employees of the University and outsiders based on a preliminary screening test. The patients were informed before the study orally and in a written form at a volunteer meeting and every volunteers signed the ICFs. Within 21 days prior to the start of the clinical study, the volunteers were taken under physical and ECG examination, respectively. Routine clinical laboratory tests, including Human Immunodeficiency Virus (HIV), Hepatitis B Virus Surface Antigen (HBsAG), drugs of abuse and pregnancy tests were also performed. Thirteen volunteers were enrolled in this pilot study: 6 males and 7 females, of which 2 males in Placebo and 4 males in TFG-treated group, accordingly 3 females in Placebo and 4 females in TFG-treated group. The age of the volunteers ranged from 23 to 55 years (Mean = 30.07, SD = 10.06). In the Placebo group the age of probands ranged from 23 to 37 years (Mean = 28.00; SD = 5.57) and in the TFG treated group from 31 to 55 years (Mean = 42.75; SD = 8.86). The BMI of the volunteers ranged between 19.89 and 31.62 (Mean = 25.80). In the Placebo group the BMI of probands ranged from 19.89 to 28.76 (Mean = 23.75; SD = 3.38) and in the TFG treated group from 22.68 to 31.62 (Mean = 27.08; SD = 2.07). The acceptance of volunteers was based on the inclusion and exclusion criteria, and were randomly separated into placebo (5) and treatment (8) groups using a computer-generated randomization code.

### 4.4. Inclusion and Exclusion Criteria

Healthy, non-smoker and non-pregnant, 18–60 years old volunteers, with BMI (body mass index) = 18.5–30 kg/m^2^, normal ECG and vital signs were included. Patients with asthma, allergic reactions, cardiovascular, respiratory, kidney, liver, endocrine and neurological diseases or psychiatric disorders in the medical history were excluded from the study. Other exclusion criteria included smoking, alcoholism, pregnancy, lactation, viral infections, medical therapy in the last 2 weeks, participation in another clinical study in the last 30 days.

### 4.5. Used Products

Test product: Dr. MAKAI Fenugreek, with active ingredient: Trigonella foenum-graecum, in 500 mg hard gelatin capsule. (Manufactured by: Trigonella MED Ltd., Mosonmagyaróvár, Hungary, OÉTI number: 6698/210, Expiry date: 31 August 2017).

Placebo product: Placebo, without active compound (filled with grits) in hard gelatin capsule (Manufactured by: Trigonella MED Ltd., Mosonmagyaróvár, Hungary).

For 10 days (from day 1 to 11) test or placebo product was self-administered by the subjects, 2 capsules p.o., three times a day. On the first day of the study, 2 capsules were administered, but only at noon and in the evening, while on the last day, 2 capsules were administered, only in the evening.

### 4.6. Hyperinsulinemic Euglycemic Glucose Clamp (HEGC)

The HEGC is considered the gold standard for the determination of insulin secretion and resistance. The method was described by DeFronzo et al. in 1979 [67] and was performed to estimate the whole body insulin sensitivity of the volunteers. The test was carried out on the first day prior to the treatment, and after 10 days of TFG or placebo treatment, on the eleventh day. Before HEGC, body weight and height recording and blood sampling for clinical chemistry—including insulin and MCH determination—was carried out. The principle of the method was to maintain euglycemia at clamped hyperinsulinemia. The average Glucose Infusion Rate (GIR), expressed in mg/kg/min necessary to compensate the hypoglycemic effect of the continuous insulin infusion is the accepted measuring unit for determining tissue insulin sensitivity.

All studies were performed at 7.00 a.m., after a 12 h overnight starvation. Two peripheral veins were cannulated for both forearms: one antecubital vein to administer the continuous glucose and insulin infusions, and one forearm vein on the contralateral arm for blood sample collection.

Insulin infusion was prepared as follows: in a 50 mL syringe, 48 mL of physiological saline, 8× BSA units of Humulin R, Eli Lilly insulin, and 2 mL of the volunteer’s own blood (to prevent insulin adhesion) were homogenized and administered via a precision perfusion pump to the volunteers. The priming infusion rate was 60 mL/h for 4 min, then 30 mL/h for further 4 min, followed by a maintenance rate of infusion that was 15 mL/h (corresponding to 40 mU/m^2^/min of insulin) administered for 112 min. The rate of the 20% dextrose infusion was adjusted to maintain the target blood glucose levels at 5.5 ± 0.5 mmol/L (euglycemic range).

Blood glucose levels were determined every 5 min using Accu-Check blood glucose meter (Accu-Check, Roche Diagnostics, Budaörs, Hungary), utilizing the glucose oxidase method, and the glucose infusion rate was adjusted according to the blood glucose level.

In a “steady state” condition, that is set during HEGC, usually in the 90th minute, the blood glucose levels are stabilized, so the rate of glucose infusion no longer requires modification, or needs to be minimally adjusted. The steady state glucose infusion rate (in the last 30 min) was used to characterize insulin sensitivity [67].

After cessing the insulin infusion, the volunteers were strictly monitored, blood glucose levels were checked, and glucose infusion was administered in case of necessity.

An intensive care expert team was ready to treat any unwanted effects with special regards to events at risk of hypoglycemia during and following the glucose clamping.

Following HEGC, post-study laboratory investigations were performed within 24 h after the last treatment phase, and there was a closing visit after 30 days.

### 4.7. Analytical Methods

The laboratory parameters, including the liver function tests, kidney function tests, electrolyte determinations, hematology, urinalysis, fasting glucose and insulin (0 min), repeated glucose and insulin (60 min, 90 min, 120 min) determinations were performed by the Department of Laboratory Medicine, University of Debrecen.

The MCH concentration was determined from blood samples separately taken into 10 mL EDTA (ethylenediamine-tetraacetic acid)-containing tubes, at 0 and 120 min on the 1st and the 11th days using a novel MCH radioimmunoassay developed by the researchers of the Department of Pharmacology and Pharmacotherapy, University of Debrecen.

### 4.8. Measurement of Melanin-Concentrating Hormone

The EDTA-tubes were centrifuged (4000 rpm, 4 °C, 15 min), and the supernatants were further processed for radioimmunoassay (RIA) analysis of MCH-like immunoreactivity (MCH-LI). The collected plasma samples were stored at −80 °C until RIA determination.

Due to the low circulating levels of MCH in plasma, a minimum of 1 mL plasma was required for the accurate determination by radioimmunoassay. Collected plasma samples were thawed, and during extraction procedures, samples and extraction reagents were stored in an ice water bath. Extraction was performed in the polypropylene RIA tubes (5 mL, 12 × 75 mm). For precipitation of plasma proteins, 1 mL plasma was added to 3 mL 96% ethyl alcohol. After mixing, the samples were incubated in an ice bath for 30 min. After centrifugation (4000 rpm, 4 °C, 10 min) the supernatants were decanted into RIA tubes and evaporated to dryness using nitrogen stream without heat. The dried samples were stored at −80 °C prior to RIA determination. In the RIA process the samples were re-dissolved in 800 µL assay buffer and assayed directly in these tubes. In our RIA examination MCH specific antiserum (MCH1/5) was applied, which was raised against a conjugate of rat MCH and bovine serum albumin (BSA) coupled by glutaraldehyde in rabbits. Rat MCH peptide was used as a RIA standard. The range of concentration was between 0 and 200 fmol/mL. Mono-125I-labeled MCH was applied as a RIA tracer prepared in our isotope laboratory. Our assay buffer (0.05 mol/L, pH 7.4 phosphate buffer) contained 0.1 M NaCl, 0.05% NaN3, 0.25% bovine serum albumin (BSA, Sigma-Aldrich, St. Louis, MO, USA). The 1 mL incubation mixture contained 100 µL MCH standards, 100 µL antiserum (working dilution 1:3500), 100 µL RIA tracer (3000 cpm/tube) and the assay puffer. After 48 h incubation at 4 °C the antibody-bound peptide was separated from the free peptide by addition of 100 µL separating suspension (10 g charcoal, 1 g dextran and 0.5 g commercial fat-free milk powder in 100 mL distilled water). After centrifugation (4000 rpm, 4 °C, 20 min) the tubes were gently decanted. Radioactivity of the precipitates was measured in a NZ310 type gamma counter (Gamma, Budapest, Hungary). The MCH-LI of the unknown samples was read from the calibration curve.

Antiserum “MCH1/5”, used in the assay turned out to be C-terminal specific without affinity for the structurally similar peptides. The average ID50 value of the calibration curves was 11.93 ± 1.78 fmol/mL, determined in ten consecutive assays. Detection limit of the assay for rat MCH was 0.2 fmol/mL. Intra-assay and inter-assay coefficients of variation were 6.84% and 9.32%, respectively [35].

### 4.9. Assesment of Insulin-Sensitivity

Based on the raw data obtained using the HEGC method we can calculate the GIR, the metabolic clearance rate of insulin (MCRI), the insulin sensitivity index (ISI) and the quantitative insulin sensitivity check index (QUICKI) that are indicators of the whole body insulin sensitivity [68,69,70].

Formulas for calculations:

GIR = glucose infusion rate [(µL/min)/BW (body weight in kg) × 100] × (20 × 10)
MCRI = (insulin infusion rate/steady state plasma insulin concentration − basal plasma insulin concentration) × 1000, expressed in mU/m^2^/min.
ISI = (glucose infusion rate/steady state plasma insulin concentration) × 100, expressed in mg/kg/min/mU/mL.
QUICKI = 1/logInsuline(0 min) (µIU/mL) + logGlucose(0 min) (mg/dL).

To estimate insulin resistance and pancreatic β-cell functions we used the widely approved homeostatic model of assessment [69,70,71,72,73].

The HOMA-IR (HOMA for insulin resistance) = (FPI × FPG)/22.5 and the HOMA-B (HOMA of β-cell function) = (20 × FPI)/(FPG − 3.5) were calculated as proposed by [72,74], where FPI = fasting plasma insulin (μIU/mL) and FPG = fasting plasma glucose (mmol/L).

### 4.10. Data Analysis and Statistics

Statistical analysis was carried out using the SAS system, version 9.2 (SAS Institute, Cary, NC, USA). The safety laboratory parameters were statistically analyzed by repeated measures ANOVA. The efficacy parameter for insulin sensitivity (GIR) for the fenugreek and placebo treatment groups was statistically analyzed by the repeated measures ANOVA in conjunction with Tukey’s HSD. (GIR was also separately analyzed with paired Student’s *t*-test). The results were considered significantly different if *p* ≤ 0.05. To analyze the association between MCH vs. glucose, insulin and serum lipid (total cholesterol, LDL-cholesterol, HDL-cholesterol and triglycerides) levels, “correlation” and “linear regression” statistical methods were used.

## 5. Conclusions

Oral preparation of fenugreek seeds improves glucose metabolism by its insulin-sensitizing effect and involves mechanisms that notably include fenugreek intake-related reduction of circulating MCH levels. Since the fenugreek-associated increase in insulin-sensitivity was the most powerful in patients with the lowest baseline GIR parameters, we conclude that it would be valued to repeat these experiments on a larger number of patients who manifest T2DM. Our results presented here suggest that members of MCH-related signal transduction pathways could become new molecular targets of drug discovery and development in the future.

## Figures and Tables

**Table 1:** (**A**) Influence of TFG (*Trigonella foenum graecum*) on GIR (Glucose Infusion Rate) in the placebo- and fenugreek-treated subjects. ** represents statistically significant difference (*p* < 0.01) compared to the Day 1 TFG group; (**B**) Individual GIR values of volunteers in Placebo and TFG-treated group. GIR is expressed as mg/kg/min.

**(A)**				
**Summary**	**GIR (mg/kg/min)**	**GIR (mg/kg/min)**	**GIR (mg/kg/min)**	**GIR (mg/kg/min)**
	**Day 1** **(Placebo, *n* = 5)**	**Day 11** **(Placebo, *n* = 5)**	**Day 1** **(TFG, *n* = 8)**	**Day 11** **(TFG, *n* = 8)**
Mean	8.45	8.5	6.63	8.13 **
S.D.	3.07	2.86	3.65	3.01
**(B)**				
**Individual GIR**	**Day 1** **(Placebo, *n* = 5)**	**Day 11** **(Placebo, *n* = 5)**	**Day 1** **(TFG, *n* = 8)**	**Day 11** **(TFG, *n* = 8)**
1.	4.99	5.79	6.49	7.46
2.	6.99	6.69	2.04	4.86
3.	12.34	12.23	6.05	8.09
4.	7.0	6.9	4.39	6.96
5.	10.95	10.87	2.52	4.14
6.			10.09	10.84
7.			12.14	13.02
8.			9.34	9.45
Mean	8.454	8.496	6.633	8.125
S.D.	3.065	2.86	3.645	3.005

## Supplementary Materials

The following are available online at http://www.mdpi.com/1422-0067/19/3/771/s1.
