# Supplementary material for: Insulin-Sensitizer Effects of Fenugreek Seeds in Parallel with Changes in Plasma MCH Levels in Healthy Volunteers
Source: Int J Mol Sci. 2018 Mar 8;19(3):771. doi: 10.3390/ijms19030771 (PMC5877632; doi:10.3390/ijms19030771)
Supplement: Supplementary file 1 [file ijms-19-00771-s001.pdf]

Supplementary Material: Insulin-Sensitizer Effects of Fenugreek Seeds in Parallel with Changes in Plasma MCH Levels in Healthy Volunteers

Rita Kiss, Katalin Szabó, Rudolf Gesztelyi, Sándor Somodi, Péter Kovács, Zoltán Szabó, József Németh, Dániel Priksz, Andrea Kurucz, Béla Juhász and Zoltán Szilvássy

Table S1. Correlation between MCH levels and serum lipid levels in placebo-treated subjects.

**Summary**	**MCH vs. Cholesterol**	**MCH vs. LDL-cholesterol**	**MCH vs. HDL-cholesterol**	**MCH vs. Triglycerides**
	**Day 1, 0 min**	**Day 1, 0 min**	**Day 1, 0 min**	**Day 1, 0 min**
r	0.3615	0.7672	−0.2856	−0.3404
95% confidence interval	−0.7646 to 0.943	−0.3562 to 0.9837	−0.9328 to 0.7977	−0.9403 to 0.7744
R squared	0.1307	0.5886	0.08155	0.1159
P value	0.5499	0.1300	0.6414	0.5751
Summary	**MCH vs. Cholesterol**	**MCH vs. LDL-cholesterol**	**MCH vs. HDL-cholesterol**	**MCH vs. Triglycerides**
	**Day 11, 0 min**	**Day 11, 0 min**	**Day 11, 0 min**	**Day 11, 0 min**
r	−0.5395	−0.1861	−0.1909	−0.8048
95% confidence interval	−0.9633 to 0.6541	−0.9177 to 0.8329	−0.9185 to 0.8314	−0.9866 to 0.267
R squared	0.2911	0.03463	0.03645	0.6478
P value	0.3480	0.7644	0.7584	0.1004
Summary	**MCH vs. Cholesterol**	**MCH vs. LDL-cholesterol**	**MCH vs. HDL-cholesterol**	**MCH vs. Triglycerides**
	**Day 1, 120 min**	**Day 1, 120 min**	**Day 1, 120 min**	**Day 1, 120 min**
r	0.1884	0.04475	0.748	−0.8822
95% confidence interval	−0.8322 to 0.9181	−0.8719 to 0.8918	−0.3949 to 0.9821	−0.9922 to 0.0005115
R squared	0.03551	0.002003	0.5594	0.7782
P value	0.7615	0.9430	0.1460	0.0477
Summary	**MCH vs. Cholesterol**	**MCH vs. LDL-cholesterol**	**MCH vs. HDL-cholesterol**	**MCH vs. Triglycerides**
	**Day 11, 120 min**	**Day 11, 120 min**	**Day 11, 120 min**	**Day 11, 120 min**
r	−0.3581	0.0425	−0.5403	−0.5645
95% confidence interval	−0.9426 to 0.7663	−0.8725 to 0.8913	−0.9633 to 0.6535	−0.9658 to 0.6331
R squared	0.1282	0.001806	0.2919	0.3186
P value	0.5540	0.9459	0.3472	0.3215

Table S2. Correlation between MCH levels and serum lipids levels of fenugreek-treated subjects.

**Summary**	**MCH vs. Cholesterol**	**MCH vs. LDL-cholesterol**	**MCH vs. HDL-cholesterol**	**MCH vs. Triglycerides**
	**Day 1, 0 min**	**Day 1, 0 min**	**Day 1, 0 min**	**Day 1, 0 min**
r	−0.3643	−0.297	−0.4412	−0.4843
95% confidence interval	−0.8506 to 0.4579	−0.8283 to 0.5156	−0.8741 to 0.3824	−0.8865 to 0.3345
R squared	0.1327	0.0882	0.1946	0.2345
P value summary	0.3749	0.4750	0.2739	0.2239
Summary	**MCH vs. Cholesterol**	**MCH vs. LDL-cholesterol**	**MCH vs. HDL-cholesterol**	**MCH vs. Triglycerides**
	**Day 11, 0 min**	**Day 11, 0 min**	**Day 11, 0 min**	**Day 11, 0 min**
r	−0.638	−0.4615	−0.5029	0.3268
95% confidence interval	−0.9263 to 0.1211	−0.88 to 0.3604	−0.8916 to 0.3125	−0.4909 to 0.8384
R squared	0.4071	0.213	0.2529	0.1068
P value	0.0887	0.2497	0.2040	0.4294
Summary	**MCH vs. Cholesterol**	**MCH vs. LDL-cholesterol**	**MCH vs. HDL-cholesterol**	**MCH vs. Triglycerides**
	**Day 1, 120 min**	**Day 1, 120 min**	**Day 1, 120 min**	**Day 1, 120 min**
r	−0.638	−0.4615	−0.5029	0.3268
95% confidence interval	−0.9263 to 0.1211	−0.88 to 0.3604	−0.8916 to 0.3125	−0.4909 to 0.8384
R squared	0.4071	0.213	0.2529	0.1068
P value	0.0887	0.2497	0.2040	0.4294
Summary	**MCH vs. Cholesterol**	**MCH vs. LDL-cholesterol**	**MCH vs. HDL-cholesterol**	**MCH vs. Triglycerides**
	**Day 11, 120 min**	**Day 11, 120 min**	**Day 11, 120 min**	**Day 11, 120 min**
r	−0.6971	−0.6746	−0.489	0.3838
95% confidence interval	−0.94 to 0.01492	−0.9349 to 0.05739	−0.8878 to 0.3291	−0.4398 to 0.8568
R squared	0.4859	0.455	0.2391	0.1473
P value	0.0547	0.0665	0.2188	0.3479

Table S3. Correlation between MCH levels and serum glucose levels of placebo- and fenugreek-treated subjects.

**Summary**	**MCH vs. Glucose**	**MCH vs. Glucose**	**MCH vs. Glucose**	**MCH vs. Glucose**
	**Day 1, 0 min (TFG, n=8)**	**Day 11, 0 min (TFG, n=8)**	**Day 1, 0 min (Placebo, n=5)**	**Day 11, 0 min (Placebo, n=5)**
r	−0.3986	0.4678	−0.0741	−0.6218
95% confidence interval	−0.8613 to 0.4257	−0.3534 to 0.8818	−0.8977 to 0.8647	−0.9712 to 0.577
R squared	0.1588	0.2188	0.005492	0.3866
P value	0.3281	0.2425	0.9057	0.2628
Summary	**MCH vs. Glucose**	**MCH vs. Glucose**	**MCH vs. Glucose**	**MCH vs. Glucose**
	**Day 1, 120 min (TFG, n=5)**	**Day 11, 120 min (TFG, n=8)**	**Day 1, 120 min (Placebo, n=5)**	**Day 11, 120 min (Placebo, n=5)**
r	−0.3986	−0.08702	−0.6839	−0.806
95% confidence interval	−0.8613 to 0.4257	−0.746 to 0.658	−0.9768 to 0.5001	−0.9866 to 0.2641
R squared	0.1588	0.007573	0.4677	0.6496
P value	0.3281	0.8377	0.2029	0.0996

Table S4. Correlation between MCH levels and plasma insulin levels of placebo- and fenugreek-treated subjects.

Summary	MCH vs. Insulin	MCH vs. Insulin	MCH vs. Insulin	MCH vs. Insulin
	**Day 1, 0 min (TFG, n=8)**	**Day 11, 0 min (TFG, n=8)**	**Day 1, 0 min (Placebo, n=5)**	**Day 11, 0 min (Placebo, n=5)**
r	−0.3363	0.4678	−0.7826	0.4548
95% confidence interval	−0.8415 to 0.4828	−0.3534 to 0.8818	−0.9849 to 0.3219	−0.7139 to 0.9542
R squared	0.1131	0.2188	0.6125	0.2068
P value	0.4154	0.2425	0.1176	0.4416
Summary	**MCH vs. Insulin**	**MCH vs. Insulin**	**MCH vs. Insulin**	**MCH vs. Insulin**
	**Day 1, 120 min (Placebo, n=5)**	**Day 11, 120 min (TFG, n=8)**	**Day 1, 120 min (Placebo, n=5)**	**Day 11, 120 min (Placebo, n=5)**
r	−0.3363	−0.08702	−0.6807	0.3949
95% confidence interval	−0.8415 to 0.4828	−0.746 to 0.658	−0.9765 to 0.5046	−0.748 to 0.9472
R squared	0.1131	0.007573	0.4633	0.1559
P value	0.4154	0.8377	0.2059	0.5106

Table S5. Steady state insulin levels and insulin levels at 90 and 120 minutes.

SS Insulin (µIU/ml).

Summary	Day 1 (Placebo, n=5)	Day 11 (Placebo, n=5)	Day 1 (TFG, n=8)	Day 11 (TFG, n=8)
1.	124.6	150.4	122.2	124
2.	99.8	112.45	62.2	92.55
3.	152.5	132.55	118.25	112.75
4.	126.1	93.5	125.95	131
5.	124.6	109.75	89	71.8
6.	124.6	150.4	79.8	74.3
7.			111.1	140.8
8.			105.4	120.55
Mean	125.37	124.84	101.74	108.47
S.D.	16.69	23.36	26.29	25.31

Insulin 90 min (µIU/ml)

Summary	Day 1 (Placebo, n=5)	Day 11 (Placebo, n=5)	Day 1 (TFG, n=8)	Day 11 (TFG, n=8)
1.	122.6	144.1	118.7	117.5
2.	103.1	97.5	68.2	94
3.	135.4	113.6	119.8	118.5
4.	126.4	85.9	122.8	112.5
5.	122.6	111.6	93.3	66.7
6.			65.7	73.4

7.			107.6	131.5
8.			107.3	118
Mean	122.02	110.54	100.43	104.01
S.D.	11.80	21.87	26.35	22.83

Insulin 120 min (μIU/ml)

**Summary**	**Day 1 (Placebo, n=5)**	**Day 11 (Placebo, n=5)**	**Day 1 (TFG, n=8)**	**Day 11 (TFG, n=8)**
1.	126.6	156.7	125.7	130.5
2.	96.5	127.4	56.2	91.1
3.	169.6	151.5	116.7	107
4.	125.8	101.1	129.1	149.5
5.	126.6	107.9	84.7	76.9
6.			93.9	75.2
7.			114.6	150.1
8.			103.5	123.1
Mean	129.02	128.92	103.05	112.93
S.D.	26.11	25.00	28.18	30.02
